# Instruments for Measuring Psychological Dimensions in Human-Robot Interaction: Systematic Review of Psychometric Properties

**DOI:** 10.2196/55597

**Published:** 2024-06-05

**Authors:** Roberto Vagnetti, Nicola Camp, Matthew Story, Khaoula Ait-Belaid, Suvobrata Mitra, Massimiliano Zecca, Alessandro Di Nuovo, Daniele Magistro

**Affiliations:** 1 Department of Sport Science School of Science and Technology Nottingham Trent University Nottingham United Kingdom; 2 Department of Computing & Advanced Wellbeing Research Centre Sheffield Hallam University Sheffield United Kingdom; 3 Wolfson School of Mechanical, Electrical and Manufacturing Engineering Loughborough University Loughborough United Kingdom; 4 Department of Psychology Nottingham Trent University Nottingham United Kingdom

**Keywords:** psychometric, human-robot interaction, psychological dimensions, robot, assessment, systematic review

## Abstract

**Background:**

Numerous user-related psychological dimensions can significantly influence the dynamics between humans and robots. For developers and researchers, it is crucial to have a comprehensive understanding of the psychometric properties of the available instruments used to assess these dimensions as they indicate the reliability and validity of the assessment.

**Objective:**

This study aims to provide a systematic review of the instruments available for assessing the psychological aspects of the relationship between people and social and domestic robots, offering a summary of their psychometric properties and the quality of the evidence.

**Methods:**

A systematic review was conducted following the PRISMA (Preferred Reporting Items for Systematic Reviews and Meta-Analyses) guidelines across different databases: Scopus, PubMed, and IEEE Xplore. The search strategy encompassed studies meeting the following inclusion criteria: (1) the instrument could assess psychological dimensions related to social and domestic robots, including attitudes, beliefs, opinions, feelings, and perceptions; (2) the study focused on validating the instrument; (3) the study evaluated the psychometric properties of the instrument; (4) the study underwent peer review; and (5) the study was in English. Studies focusing on industrial robots, rescue robots, or robotic arms or those primarily concerned with technology validation or measuring anthropomorphism were excluded. Independent reviewers extracted instrument properties and the methodological quality of their evidence following the Consensus-Based Standards for the Selection of Health Measurement Instruments guidelines.

**Results:**

From 3828 identified records, the search strategy yielded 34 (0.89%) articles that validated and examined the psychometric properties of 27 instruments designed to assess individuals’ psychological dimensions in relation to social and domestic robots. These instruments encompass a broad spectrum of psychological dimensions. While most studies predominantly focused on structural validity (24/27, 89%) and internal consistency (26/27, 96%), consideration of other psychometric properties was frequently inconsistent or absent. No instrument evaluated measurement error and responsiveness despite their significance in the clinical context. Most of the instruments (17/27, 63%) were targeted at both adults and older adults (aged ≥18 years). There was a limited number of instruments specifically designed for children, older adults, and health care contexts.

**Conclusions:**

Given the strong interest in assessing psychological dimensions in the human-robot relationship, there is a need to develop new instruments using more rigorous methodologies and consider a broader range of psychometric properties. This is essential to ensure the creation of reliable and valid measures for assessing people’s psychological dimensions regarding social and domestic robots. Among its limitations, this review included instruments applicable to both social and domestic robots while excluding those for other specific types of robots (eg, industrial robots).

## Introduction

### Background

There is a growing interest in the field of social robotics when it comes to creating robots that can cater to people’s needs. This is evidenced by the increasing number of publications covering various aspects of robotics [1]. This interest stems from the desire to develop robots that can engage in social interaction with humans, serving as collaborators, companions, tutors, and partners in various applications. Applications of social and domestic robots cover widespread areas; for instance, they have been proposed for educational purposes [2], for mental health and well-being [3], to support older adults in their homes [4-6], or to support different clinical populations such as people with autism spectrum disorder [7] or dementia [8].

While many studies have explored users’ opinions and requirements to design and develop this technology to meet their needs in a participatory design framework [9-13], a major challenge for the success of social robots is the fact that their mere presence in everyday life does not automatically increase their chances of being accepted or users’ willingness to interact with them [14]. Thus, understanding the perspectives and preferences of people regarding robots represents a crucial point for their development and acceptance [15-17]. How a robot is perceived plays a major role in the human-robot relationship [18]. Existing literature has identified several factors linked to individuals’ predispositions toward robots and how robots are used [19]. Peoples’ robot acceptance is influenced by attitudes and intentions to use robots [20]. According to the Unified Theory of Acceptance and Use of Technology [21], factors in the intention to use robots include attitude, perceived usefulness, perceived ease of use, enjoyment, trust, and anxiety. However, many other psychological dimensions have been investigated within the human-robot relations, such as beliefs [22], adaptability, control, companionship, sociability [19], attractiveness [23], social presence [24], intentionality [25], and expectations [26]. Thus, numerous user-related psychological dimensions can significantly influence the dynamics of the human-robot relationship. Systematic reviews focusing on different dimensions related to human-robot interactions with social robots reportedly indicate that most of the assessments are made using self-report measurements, raising concerns about their suitability [27-29].

For developers and researchers, it is crucial to have a comprehensive understanding of the psychometric properties of the available instruments. To make a reasoned decision regarding the use of instruments in research, it is crucial to possess an understanding of instrument properties and make comparisons between them [30]. Indeed, psychometric properties encompass attributes of an instrument that serve as indicators of its reliability and validity [31]. They help ascertain whether the measure accurately assesses what it is meant to assess and consistently gauges the intended dimension. In this context, systematic reviews of instrument psychometric properties can assist practitioners and researchers in choosing the most suitable measurement instrument tailored to their specific needs [32]. These reviews are valuable because they consider both the instrument psychometric properties and the methodological quality of the studies conducted to assess them [33]. This knowledge is essential for making informed decisions and effectively evaluating the performance and impact of robots in various applications.

### Objectives

The aim of this research was to conduct a systematic review of the instruments documented in the literature for assessing individuals’ psychological dimensions in relation to social and domestic robots, such as attitudes, beliefs, perceptions, opinions, and emotions. In this review, *instrument* refers to a specific tool used for data collection and measurement, such as questionnaires, scales, and interviews. This review assessed both the instrument psychometric properties and the quality of evidence linked to each property with a view to (1) provide practitioners and researchers with a comprehensive guide to the available instruments and their psychometric properties, enabling them to make informed choices based on their specific requirements; and (2) establish indications for the future development and validation of such instruments.

## Methods

### Search Strategy and Eligibility Criteria

A systematic review was conducted following the PRISMA (Preferred Reporting Items for Systematic Reviews and Meta-Analyses) guidelines [34]. The search was conducted from June 2023 to July 2023 in the following computerized databases: Scopus, PubMed, and IEEE Xplore. This was done as searching at least 2 databases is recommended for the best coverage of the topic and to decrease chances of inappropriate conclusions [35]. The search strategy aimed to find literature related to the validation and assessment of the psychometric properties of instruments designed to evaluate individuals’ psychological dimensions in relation to robots. To accomplish this, the following index terms—“robot*,” “social,” “home,” “domestic,” “questionnaire,” “survey,” “assessment,” “measure*,” “psychom*,” “valid,” and “reliab*”—were used. The search strategy is provided in Multimedia Appendix 1.

The search strategy aimed to incorporate instruments suitable for use with social and domestic robots. Therefore, those specifically designed for other types of robots were excluded. The search strategy targeted studies meeting the following inclusion criteria: (1) the instrument could assess psychological dimensions related to social and domestic robots, including attitudes, beliefs, opinions, feelings, and perceptions; (2) the study focused on validating the instrument; (3) the study evaluated the psychometric properties of the instrument; (4) the study underwent peer review; and (5) the study was in English. Given the focus of our research, studies centered on industrial robots, rescue robots, or robotic arms or those primarily validating technology or measuring anthropomorphism were excluded. In total, 3 members of the research group independently assessed the eligibility of the articles after establishing the criteria with the research team. Initially, the titles and abstracts of the articles resulting from the search were screened based on the established criteria. Those that passed the screening were then evaluated through full-text reading. At the end of each step (title and abstract screening and full-text screening), interrater agreement among the 3 reviewers was evaluated, indicating good agreement (Fleiss κ=0.83 and 0.92, respectively), and any disagreements were resolved through discussion. The systematic review and protocol were not registered with any relevant database.

### Data Extraction

From the included studies, the following data were extracted for each instrument: the name of the scale; references identified during the systematic review process; the total number of items; a description of the type of items; a description of the construct measured; a description of the subscales, if any; the number of items in each subscale; the administration of the instrument; the target population of the instrument; and the characteristics of the population used to validate the instrument, including nationality, sample sizes, and age (mean, SD, and range). A total of 2 reviewers extracted these data independently, and any disagreements were resolved through consensus with a third reviewer.

### Assessment of the Instrument Properties and Methodological Quality

The Consensus-Based Standards for the Selection of Health Measurement Instruments (COSMIN) guidelines were adapted to evaluate instrument properties and the methodological quality of the evidence obtained from the identified studies [36-38]. The instrument properties defined and considered by the COSMIN guidelines include content validity (which assesses item relevance, comprehensiveness, and comprehensibility), structural validity, internal consistency, cross-cultural validity, measurement invariance, reliability, measurement error, criterion validity, construct validity, and responsiveness.

The overall rating of each psychometric property per instrument could be sufficient (+), insufficient (−), indeterminate (?), or inconsistent (±) depending on the scores obtained across all the studies for that given measure. While “sufficient” or “insufficient” clearly indicate whether the criteria were met, the procedure considered studies as “indeterminate” when they addressed the relevant aspect but failed to provide sufficient information to determine whether the criteria had been met [36,38]. Furthermore, the “inconsistent” category encompassed a combination of both “sufficient” and “insufficient” results. We chose not to resolve inconsistent results but, instead, to provide this process to individuals interested in using the reviewed instruments, considering this study a valuable summary of the instrument properties available to date. In the context of content validity evaluations, “insufficient” was assigned to each subcategory (relevance, comprehensiveness, and comprehensibility) when these aspects were not evaluated during the development or validation of the measure. In terms of hypothesis testing for construct validity, and considering the multiple dimensions assessed using the identified measures, each study was independently evaluated based on the following suggested generic hypothesis [36]: when the instruments measure related but dissimilar constructs, correlations should fall within the range of 0.30 to 0.50, and when they measure similar constructs, the correlations should be ≥0.50. We considered group differences when hypotheses were clearly stated, supported by the literature, and used specifically to assess instrument properties. An important aspect to consider is that, due to the various constructs that the instrument could assess and the relatively recent development of these scales, it is challenging to establish a reliable gold standard. Consequently, this study did not assess criterion validity among the indicators used to validate the instruments.

The methodological quality of each instrument property in each study was assessed as “high,” “moderate,” “low,” or “very low” following the COSMIN guidelines [36-38]. Subsequently, the overall quality of the body of evidence for each psychometric property could be downgraded based on 3 factors: risk of bias, inconsistency of findings (less relevant for content validity), and imprecision (low sample sizes). It is worth noting that “indirectness” was not evaluated because the review lacked a defined target population.

In total, 2 independent raters extracted data from each record included in the research process and assessed the risk of bias for each psychometric property in each study. Any disagreements that arose were resolved through consensus with a third reviewer.

## Results

### Overview

The search strategy resulted in a total of 3828 articles. After the removal of 14.52% (556/3828) of duplicates, a further 83.49% (3196/3828) of the articles were excluded during title and abstract screening. Then, of the remaining 76 articles, 42 (55%) were excluded during the full-text evaluation for not meeting the research criteria.

**Figure 1:**
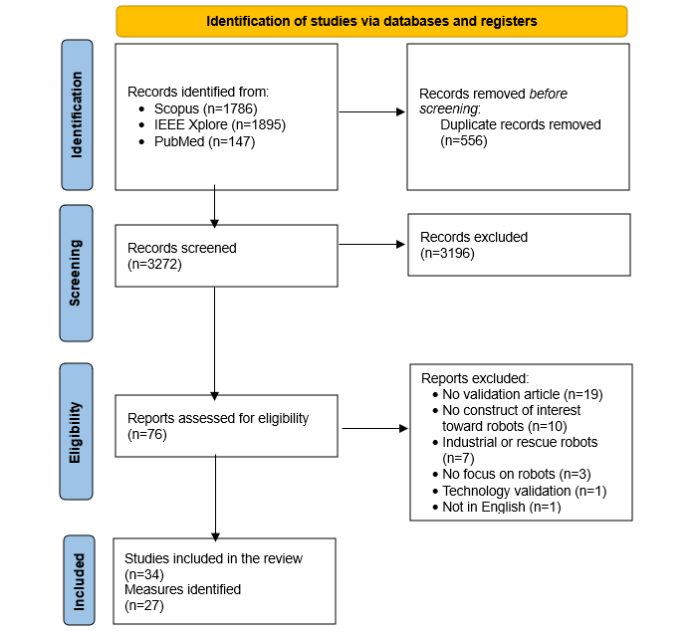
Flowchart of the search and study selection.

The overall search strategy resulted in the inclusion of a total of 34 articles evaluating 27 measures aimed at assessing people’s attitudes toward social robots. A summary of the research process is provided in Figure 1. The information extracted for each measure is reported in Table 1, whereas ratings and quality of evidence are reported in Table 2. In the following sections, we discuss the results per instrument, grouping them according to the included population.

**Table 1:** Summary of the data extracted for the instruments identified through the systematic review.

Instrument name	Study	Total items	Type of items	Construct or constructs	Subscales (number of items)	Type of administration	Target population	Validation population nationality and number of participants	Age of the validation population (y)
COIRS^a^	Robert and van den Bergh [39]	12	Scale from 0 to 4	Assess openness to robot interaction	Intrinsic interest in interacting with a robot (3); openness to socioemotional interactions with a robot (5); openness to utilitarian interaction with a robot (4)	Self-report	Children	American	Range 8-11
Robot gratification questionnaire^b^	de Jong et al [40]	26	5-point Likert scale	Assess gratification (sought and obtained)	Hedonic gratification—sought (3); hedonic gratification—obtained (3); informative gratification—sought (3); informative gratification—obtained (3); social gratification—obtained (3); social gratification—sought (3); experiential gratification—sought (4); experiential gratification—obtained (4)	Observational	Children	Dutch—24 children (15 male; 9 female)	Mean 9.31 (SD 1.15; range 7-11)
Robot social presence scale^b^	Chen et al [41]	17	5-point Likert scale	Assess robot social presence	Perceived presence (4); interaction behavior perception (4); interactive expression and information understanding (4); perceived emotional interdependence (4); attention allocation (4); emotional understanding and expressiveness (4)	Self-report	General population	Chinese—494 (174 male; 320 female)	Range 18-60
REI^c^	Bevilacqua et al [42]	41	5-point Likert scale	Assess the acceptability of SARs^d^ in the older adult population	Perceived robot personality (11); human-robot interaction (10); perceived benefit (6); easiness of use (6); perceived usefulness (7)	Self-report	Older adults	Not reported—21 (13 male; 8 female)	Mean 62.9 (SD 3.9)
UNRAQ^e^	Tobis et al [43,44]	34	5-point Likert scale	Assess older persons’ needs and requirements regarding the properties and functions of a robot	Interaction with the robot and technical issues (10); assistive role of the robot (13); social aspects of using the robot (6); ethical issues (5)	Self-report	General population	Not reported—720 (179 male; 541 female)	Mean 52.0 (SD 37.0; range 19-91)
A revision of the TSES-R^f^	Zhang et al [45]	20	5-point Likert scale	Parental expectations regarding robots for health care	Capabilities dimension (5); fictional view dimension (5); social or emotional dimension (4); playful distraction dimension (3); assistive role dimension (3)	Self-report	Parents	Not reported	Not reported
GAToRS^g^	Koverola et al [46]	20	7-point Likert scale	People’s attitudes toward robots	Personal-level positive attitude (5); personal-level negative attitude (5); societal-level positive attitude (5); societal-level negative attitude (5)	Self-report	General population	Finnish—477 (192 male; 283 female)	Mean 40.23 (SD 13.51); all participants were aged >18 years
SPRS^h^	Mandl et al [47]	18	Semantic differential scale	Measure social perception of robots	Anthropomorphism (6); morality or sociability (6); activity or cooperation (4)	Self-report	General population	German, Austrian, Swiss, and from other countries—1032 (538 male; 480 female; 14 nonbinary)	Mean 33.83 (SD 12.66)
SSRIT^i^	Chi et al [48]	50	5-point Likert scale	Assess trust in interactions with AI^j^ social robots in service delivery	Familiarity (4); robot use self-efficacy (5); social influence (4); technology attachment (3); trust stance in technology (3); anthropomorphism (7); robot performance (9); effort expectancy (4); perceived service risk (5); robot service fit (3); facilitating robot use condition (3)	Self-report	Consumers	Ethnicities were reported—sample 1: 452 (38.9% male; 60.8% female; 0.2% other); sample 2: 362 (38.7% male; 61% female; 0.3% other)	Range >18->65
ATTUSR-C^k^	Chen et al [49]	15	5-point Likert scale	Assess ATTUSR-C questionnaire for use with Taiwanese health personnel	Unidimensional	Self-report	Health personnel	Taiwanese—95 (95% female)	Mean 44.5 (SD 11.9; range 25-63)
Intentional Acceptance of Social Robots	de Jong et al [50]	4	5-point bar scale	Assess children’s intention to use social robots	Unidimensional	Self-report	Children	Dutch—87 (39 male; 48 female)	Mean 9.17 (SD 0.85; range 7-11)
ERAS^l^	Sisman et al [51]	17	5-point Likert scale	Measure the attitudes of school students toward the use of humanoid robots in educational settings	Engagement (5); enjoyment (4); anxiety (4); intention (4)	Self-report	Children	Not reported—232 (128 male; 104 female)	Range 10-13
SE-HRI^m^	der Pütten and Bock [52]	18	6-point Likert scale	Measure people’s perceived self-efficacy in dealing with robots	Unidimensional	Self-report	General population	German—450 (288 female; 4 unknown); American—209 (104 male; 105 female)	German: mean 25.15 (SD 6.66; range 18-59); American: mean 26.48 (SD 9.11; range 16-69)
SE-HRI short version	der Pütten and Bock [52]	10	6-point Likert scale	Measure people’s perceived self-efficacy in dealing with robots	Unidimensional	Self-report	General population	English-speaking and German-speaking sample—196 (101 male; 95 female)	Mean 36.91 (SD 13.97; range 18-82)
MCRS^n^	Nomura et al [53]	21	7-point Likert scale	Measure moral concern for robots	Basic moral concern (12); concern for psychological harm (9)	Self-report	General population	Japanese—group 1: 121 (66 male; 55 female); group 2: 200 (100 male; 100 female)	Mean 20.1 (SD 1.6) for group 1; range 20s-60s for group 2
RoSAS^o^	Carpinella et al [54] and Oliveira et al [55]	18 (note: 10 for Portuguese version)	9-point Likert scale	Measure social perception of robots	Warmth (6); competence (6); discomfort (6)	Self-report	General population	Not reported—210 (105 male; 104 female; 1 not identified); Portuguese—185 (45% male; 55% female)	Not reported—Portuguese sample: mean 23.40 (SD 5.21; range 18-35)
HEXACO-60 for HRI^b,p^	Siri et al [56]	60	5-point Likert scale	Evaluate how people perceive the personality traits of robots	Empathy, altruism, or sociability; integrity; dependability; self-confidence	Self-report	General population	Italian—133 (not reported)	Mean 34.46 (SD 14.17; range 19-65)
Sense of safety and security for robots in elder care^b^	Akalin et al [57]	12	Semantic difference scales	Measure sense of safety and security for robots in eldercare	Sense of safety (6); sense of security (6)	Self-report	General population	Not reported—100 (47 male; 53 female)	Mean 35.48 (SD 10.58; range 14-62)
PERNOD^q^	Kamide et al [58]	33	7-point Likert scale	Evaluate humanoid robots	Familiarity (12); utility (7); motion (4); controllability (5); toughness (5)	Self-report	General population	Japanese—380 university students (140 male; 239 female; 1 unknown)	Mean 20.31 (SD 2.89)
Multidimensional Robot Attitude Scale	Ninomiya et al [59]	49	7-point Likert scale	Assess attitudes toward domestic robots	Familiarity (6); interest (7); negative attitude (5); self-efficacy (4); appearance (7); utility (5); cost (3); variety (3)	Self-report	Adults	Japanese—175 (77.8% male); Taiwanese—130 (46.9% male); Chinese—40.5% male	Japanese: mean 22.3 (SD 1.9); Chinese: mean 23.6 (SD 1.6); Taiwanese: mean 24.2 (SD 5.0)
RUSH^r^	Turja et al [60]	6	5-point Likert scale	Measure robot use self-efficacy in health care work	Unidimensional	Self-report	Health care workers	Finnish—3 samples: 200 home care workers (93.5% female), 1889 nurses (89.8% female),and 1554 nurses and physiotherapists (95% female)	Sample 1: mean 43.2 (SD 11.8; range 19-65); sample 2: mean 45.5 (SD 12.1; range 17-68); sample 3: mean 47.5 (SD 10.4; range 19-70)
RAS^s^	Nomura et al [61] and Cai et al [62]	11	6-point Likert scale	Measuring the anxiety that prevents individuals from interacting with robots that have functions of communication in daily life	Anxiety toward communication capability of robots (3); anxiety toward behavioral characteristics of robots (4); anxiety toward discourse with robots (4)	Self-report	General population	Japanese: 400 university students (197 male; 199 female; 4 unknown); Chinese: sample 1 composed of 305 adults (138 male; 167 female) and sample 2 composed of 740 adults (319 male; 421 female)	Japanese: mean 21.4; Chinese: sample 1 range 18-≥60 and sample 2 range 18-60
RERS^t^	Nomura and Kanda [63]	18	7-point Likert scale	Measure people’s expectations for rapport	Expectation as a conversation partner (11); expectation for togetherness (7)	Self-report	General population	Not reported—2s0 university students (not reported)	Not reported
Child-robot relationship formation^b^	Straten et al [64]	13	5-point bar scales	Assess child-robot relationship formation	Closeness (5); trust (4); perceived social support (5)	Self-reported	Children in middle childhood	Dutch—87 children (39 male; 48 female)	Mean 9.17 (SD 0.85; range 7-11)
Almere model	Heerink et al [21] and He et al [65]	41 (30 for the Mandarin version)	5-point Likert scale	Acceptance of assistive social agents by older adults	Anxiety (4); attitude toward the assistive social agent (3); facilitating conditions (2); intention to use (3); perceived adaptiveness (3); perceived enjoyment (5); perceived ease of use (5); perceived sociability (4); perceived usefulness (3); social influence (2); social presence (5); trust (2)	Self-reported	Older adults	Dutch—experiment 1: 40 older adults (18 male; 22 female), experiment 2: 88 participants (28 male; 60 female), experiment 3: 30 older adults (8 male; 22 female), and experiment 4: 30 older adults (16 male; 14 female); Chinese—317 (55.5% female)	Dutch—experiment 1: range 65-89, experiment 2: NA^u^, experiment 3: range 65-94, and experiment 4: range 65-89; Chinese—mean 70.3 (SD 7.5)
Frankenstein Syndrome Questionnaire	Nomura et al [66,67]	30	7-point Likert scale	Measure acceptance of humanoid robots, including expectations and anxieties regarding this technology in the public	General anxiety toward humanoid robots (13); apprehension toward social risks of humanoid robots (5); trustworthiness for developers of humanoid robots (4); expectation for humanoid robots in daily life (5)	Self-reported	General population	Japanese (the questionnaire is also available in English)—1000 (500 male; 500 female)	Range 20s-60s
NARS^v^	Nomura et al [68], Piçarra et al [69], Pochwatko et al [70], Syrdal et al [71], and Xia and LeTendre [72]	14 (the Portuguese and Polish versions have 12 items, and the English version has 11 items)	5-point Likert scale (7-point Likert scale for the Portuguese and Polish versions)	Measure humans’ negative attitudes toward robots	Negative attitudes toward situations of interaction with robots (6); negative attitudes toward the social influence of robots (5); negative attitudes toward emotions in interaction with robots (3; note: the Portuguese and Polish versions have 2 dimensions, NARHT^w^ and NATIR^x^, and the English version has 3 dimensions measuring different constructs)	Self-reported	General population	Japanese—240 university students (146 male; 92 female; 2 unknown); American—54 undergraduate students (13 male; 41 female); Portuguese—4 studies with a total sample of 997 (401 male; 598 female; 3 not reported); Polish—213 (80 male; 91 female; 42 not reported); English—28 university students and staff (14 male; 14 female)	Japanese—mean 22.0 (SD not reported); American—range 18-25; Portuguese—range 18-71; Polish—mean 29.36 (SD 10.15); English—range 18-55

^a^COIRS: Children’s Openness to Interacting With a Robot Scale.

^b^The name of the instrument was not provided in the original article.

^c^REI: Robot-Era Inventory.

^d^SAR: socially assistive robot.

^e^UNRAQ: Users’ Needs, Requirements, and Abilities Questionnaire.

^f^TSES-R: Technology-Specific Expectation Scale–R.

^g^GAToRS: General Attitudes Toward Robots Scale.

^h^SPRS: Social Perception of Robots Scale.

^i^SSRIT: Social Service Robot Interaction Trust.

^j^AI: artificial intelligence.

^k^ATTUSR-C: Chinese version of the Attitudes Toward The Use of Social Robots.

^l^ERAS: Educational Robot Attitude Scale.

^m^SE-HRI: Self-Efficacy in Human-Robot Interaction Scale.

^n^MCRS: Moral Concern for Robots Scale.

^o^RoSAS: Robotic Social Attributes Scale.

^p^HRI: human-robot interaction.

^q^PERNOD: Perception to Humanoid scale.

^r^RUSH: Robot Use Self-Efficacy in Healthcare Work.

^s^RAS: Robot Anxiety Scale.

^t^RERS: Rapport-Expectation With a Robot Scale.

^u^Not available.

^v^NARS: Negative Attitudes Toward Robots Scale.

^w^NARHT: negative attitudes toward robots with human traits.

^x^NATIR: negative attitudes toward interactions with robots.

**Table 2:** Results obtained from the overall rating (OR) quality of evidence (QoE)^a^.

Instrument	Content validity								Structural validity		Internal consistency		Cross-cultural validity		Measurement invariance		Reliability		Construct validity	
	Overall		Relevance		Comprehensiveness		Comprehensibility		OR	QoE	OR	QoE	OR	QoE	OR	QoE	OR	QoE	OR	QoE
	OR	QoE	OR	QoE	OR	QoE	OR	QoE												
COIRS^b^	±^c^	Low	−^d^	Low	+^e^	Low	+	Low	+	High	+	High	—^f^	—	—	—	—	—	?^g^	Low
Robot gratification questionnaire^h^	±	Low	+	Low	−	Low	−	Low	?	Low	±	Low	—	—	—	—	—	—	±	Very low
Robot social presence scale^h^	+	Moderate	+	Moderate	+	Moderate	+	Moderate	+	Moderate	+	High	—	—	—	—	—	—	—	—
REI^i^	−	Very low	±	Low	−	Very low	−	Very low	?	Very low	−	Very low	—	—	—	—	—	—	?	Very low
UNRAQ^j^	−	Very low	−	Very low	−	Very low	−	Very low	—	—	±	High	—	—	—	—	+	Moderate	—	—
TSES-R^k^	−	Very low	−	Very low	−	Very low	−	Very low	?	Very low	+	Moderate	—	—	—	—	—	—	—	—
GAToRS^l^	±	Very low	+	Low	−	Very low	−	Very low	+	High	+	High	—	—	—	—	—	—	±	High
SPRS^m^	−	Very low	−	Low	−	Very low	−	Very low	−	High	±	High	—	—	—	—	—	—	±	High
SSRIT^n^	+	Moderate	+	Moderate	+	Moderate	+	Low	+	High	+	High	—	—	—	—	—	—	+	High
ATTUSR-C^o^	±	Very low	+	Very low	−	Very low	+	Very low	?	Moderate	+	Moderate	—	—	—	—	—	—	—	—
Intentional Acceptance of Social Robots	−	Very low	−	Very low	−	Very low	−	Low	+	Moderate	+	Moderate	—	—	+	Low	—	—	±	Very low
ERAS^p^	−	Moderate	−	Moderate	−	Moderate	+	Moderate	?	High	+	High	—	—	—	—	—	—	—	—
SE-HRI^q^	±	Very low	+	Very low	−	Very low	+	Very low	+	High	+	High	—	—	—	—	—	—	+	High
SE-HRI (short version)	—	—	—	—	—	—	—	—	—	—	+	High	—	—	—	—	—	—	+	High
MCRS^r^	−	Very low	−	Very low	−	Very low	−	Very low	?	Moderate	+	High	—	—	—	—	—	—	?	Very low
RoSAS^s^	−	Very low	−	Very low	−	Very low	+	Low	+	High	+	High	—	—	—	—	±	Moderate	±	Moderate
HEXACO-60 for HRI^d^^,t^	−	Very low	±	Very low	−	Very low	−	Very low	?	Very low	—	—	—	—	—	—	—	—	—	—
Sense of safety and security for robots in elder care^h^	−	Very low	−	Very low	−	Very low	−	Very low	?	Very low	+	Moderate	—	—	—	—	—	—	—	—
PERNOD^u^	−	Very low	−	Low	−	Very low	−	Very low	?	High	+	High	—	—	—	—	—	—	—	—
Multidimensional Robot Attitude Scale	−	Very low	−	Very low	−	Low	−	Very low	—	—	±	High	—	—	—	—	—	—	—	—
RUSH^v^	−	Very low	−	Very low	−	Very low	−	Very low	?	High	+	High	—	—	—	—	—	—	−	Very low
RAS^w^	+	Moderate	+	Moderate	+	Moderate	?	Moderate	+	High	+	High	—	—	—	—	—	—	±	High
RERS^x^	−	Very low	±	Very low	−	Very low	−	Very low	?	Very low	+	Moderate	—	—	—	—	—	—	?	Low
Child-robot relationship formation^h^	±	Very low	±	Very low	−	Very low	+	Low	+	Moderate	+	Moderate	—	—	—	—	—	—	±	Very low
Almere model	+	Moderate	+	Moderate	+	Moderate	?	Moderate	+	High	+	High	—	—	—	—	+	High	?	Low
Frankenstein Syndrome Questionnaire	−	Moderate	−	Moderate	−	Moderate	−	Moderate	?	Moderate	+	High	−	Very low	—	—	—	—	—	—
NARS^y^	−	Low	−	Low	−	Low	−	Very low	−	High	+	High	−	Very low	—	—	±	Low	±	High

^a^Measurement error and responsiveness are absent from the table because no article assessed these properties, and criterion validity is not reported in accordance with the explanation given in the *Methods* section.

^b^COIRS: Children’s Openness to Interacting With a Robot Scale.

^c^Inconsistent rating.

^d^Insufficient rating.

^e^Sufficient rating.

^f^Blank cells represent psychometric properties that were not evaluated for that instrument.

^g^Indeterminate rating.

^h^The name of the instrument was not reported in the original article.

^i^REI: Robot-Era Inventory.

^j^UNRAQ: Users’ Needs, Requirements, and Abilities Questionnaire.

^k^TSES-R: Technology-Specific Expectation Scale–R.

^l^GAToRS: General Attitudes Toward Robots Scale.

^m^SPRS: Social Perception of Robots Scale.

^n^SSRIT: Social Service Robot Interaction Trust.

^o^ATTUSR-C: Chinese version of the Attitudes Toward the Use of Social Robots.

^p^ERAS: Educational Robot Attitude Scale.

^q^SE-HRI: Self-Efficacy in Human-Robot Interaction Scale.

^r^MCRS: Moral Concern for Robots Scale.

^s^RoSAS: Robotic Social Attributes Scale.

^t^HRI: human-robot interaction.

^u^PERNOD: Perception to Humanoid scale.

^v^RUSH: Robot Use Self-Efficacy in Healthcare Work.

^w^RAS: Robot Anxiety Scale.

^x^RERS: Rapport-Expectation With a Robot Scale.

^y^NARS: Negative Attitudes Toward Robots Scale.

### Instruments to Assess Children’s Psychological Dimensions Toward Robots

The *Children’s Openness to Interacting With a Robot Scale* [39] measures openness to new experiences and psychological boundaries related to robot interactions. The scale was developed through focus groups with parents, teachers, and researchers and underwent cognitive pretesting with colleagues and researchers. During the validation, exploratory factor analysis (EFA) revealed a 3D structure with good internal consistency (Cronbach α ranging from 0.72 to 0.78) and sufficient structural validity (root mean square error of approximation [RMSEA]=0.07; comparative fit index [CFI]=0.93; root mean square residual=0.07) for the 3 dimensions. Construct validity was assessed by correlating the average Children’s Openness to Interacting With a Robot Scale score with those of other scales. However, correlations were not performed with the subscales, making the construct validity for each subscale unclear. A comparison by age and gender found no significant differences, although the purpose of the comparison was not reported.

The questionnaire developed by de Jong et al [40] aimed to assess children’s uses of and gratifications regarding social robots based on the literature on children’s media gratifications. After a brief interaction with a social robot, 88 Dutch children were interviewed. Through coding of their responses to an open-ended question, categories of gratifications were identified, and a questionnaire was developed to measure 4 types of gratification. The items were derived from previous questionnaires and children’s answers. The gratification types were subsequently categorized into sought and obtained, although the theoretical rationale for this choice was not provided. During the validation, 2 subscales did not reach sufficient internal consistency. The EFA results did not provide information about the goodness of the 8D solution. Some of the subscales did not provide sufficient evidence for the expected hypothesis tested by the authors (Pearson correlations ranging from 0.12 to 0.78).

The *Intentional Acceptance of Social Robots* [50] is a unidimensional instrument developed to assess children’s intentional acceptance of social robots, defined as children’s intention to use a social robot repeatedly or for a long period in their daily life. The researchers reviewed existing measures and focused on the scale by Heerink et al [21]. They adjusted and refined items by referencing specific activities and adapting the language for children, through discussions, and with suggestions from primary school teachers. The items were also translated into Dutch. Pilot-testing with 4 children led to further adjustments. The confirmatory factor analysis (CFA) revealed a good fit of the data (N=87, χ^2^_2_=3.6, *P*=.16; CFI=0.97; standardized root mean square residual=0.04). Measurement invariance was assessed between boys and girls, showing sufficient results. Internal consistency showed sufficient results for the overall sample (range 0.72-0.85). According to hypothesis testing, the scale showed enough correlation with the enjoyment measure (*r*=0.49) but low correlation with other measures (ie, social presence [*r*=0.24] and social anxiety [*r*=−0.20]).

The *Educational Robot Attitude Scale* [51] was developed through a process involving the creation of an item pool based on existing literature. The scale was reviewed by experts for content and face validity. A pilot test with 20 schoolchildren was conducted to assess item comprehension. The scale showed a 4D solution according to EFA (fitting indexes were not reported). The reliability of the scale was satisfactory (Cronbach α ranged from 0.81 to 0.85).

Straten et al [64] developed a measure to assess child-robot relationships using 3 self-report scales of closeness, trust, and perceived social support in which constructs were derived from theories of interpersonal relationships. The researchers developed the scales by reviewing existing measures and refining item content translated into Dutch. Comprehensibility was assessed through teachers and pilot studies. The measure’s validation demonstrated a good model fit based on CFA results (N=87, χ^2^_62_=62.3, *P*=.47; CFI=0.999; standardized root mean square error [SRMR]=0.052). Hypothesis testing with concurrently measured variables, which were significantly shortened, yielded mixed results.

### Instruments to Assess Psychological Dimensions of Adults (Aged ≥18 Years) Toward Robots

Chen et al [41] proposed a 6D questionnaire to assess robots’ social presence. Researchers retrieved papers related to social presence and identified questions for a human-robot interaction scale divided into theoretical dimensions following expert evaluation and translation. A total of 3 experts in artificial intelligence, psychology, and sociology respectively assessed the proposed definition and model, tested face validity, and reviewed content and discriminant validity for each dimension of the scale. Then, 5 respondents experienced in using social robots were invited for structured interviews to assess the clarity, precision, repetition, conflict, and understandability of the questionnaire. Validation results indicated good fit from EFA (chi-square–to–df ratio=2.160; RMSEA=0.048; Tucker-Lewis index [TLI]=0.928; normed fit index=0.939; Adjusted Goodness of Fit Index [AGFI]=0.926; SRMR=0.052; CFI=0.966; goodness-of-fit index [GFI]=0.950), and the Cronbach α values were of >0.70.

The *Users’ Needs, Requirements, and Abilities Questionnaire* [43,44] was developed through a process that involved a literature review and collaboration with the ENRICHME project partners. It is an instrument that can be used to collect data on the use of social robots in the care of older people. The validation sample consisted of 720 older adult participants, 125 of whom repeated the assessment 2 weeks apart. Evaluation of psychometric properties indicated good Cronbach α values for each dimension (all >0.70) and test-retest reliability for each subscale measured using the intraclass correlation coefficient (range 0.81-0.93).

The *General Attitudes Toward Robots Scale* [46] was developed to assess attitude as a predisposition to respond favorably or unfavorably to objects in the world and makes a distinction between personal and societal levels of attitudes toward robots, differentiating them between positive and negative. In the pilot study, the authors only reported that the measure was developed partly based on other instruments. The 4D factor was considered adequate as it fell between the suggested factors of different evaluation methods. Only 2 of the dimensions had a Cronbach α value of >0.70. The authors developed a revised version of the questionnaire by conducting a pilot study, extracting items from other instruments, and collecting new items through open questions posted in science fiction forums and robotics-oriented Facebook groups. The authors further refined these items through various EFAs. The final version of the questionnaire consisted of 20 items along with 4 criterion items. A CFA indicated good fit (*χ^2^*_164_=430.0, *P*<.001; CFI=0.91; TLI=0.896; RMSEA=0.058, 90% CI 0.052-0.064; SRMR=0.057), and the Cronbach α values for each subscale were of >0.70. Correlations with the Negative Attitudes Toward Robots Scale (NARS) indicated mixed results (range 0.2-0.8); however, the authors did not report a specific hypothesis and, given that the General Attitudes Toward Robots Scale measures attitude toward robots, we would have expected strong correlations of >0.50.

The *Social Perception of Robots Scale* [47] was developed as a short scale for measuring social perceptions of robots that comprises sociability, competence, morality, and anthropomorphism that can be applied to different robots in diverse research settings. Although a definition for each scale was provided, the authors did not describe a theoretical background for the social perception dimension and for its subcomponents. The authors composed items based on 3 different instruments to address the 3 main dimensions of social perception. The EFA results indicated a 3D factor (anthropomorphism, morality or sociability, and activity or cooperation), and a subsequent CFA did not indicate good fit (*χ^2^*_115_=508.1, *P*<.001; RMSEA=0.101; CFI=0.796; TLI=0.759; SRMR=0.096). Regarding internal consistency, the third dimension resulted in a low index (Cronbach α=0.64), whereas the first and second dimensions had sufficient indexes (Cronbach α=0.82 and 0.85, respectively). Regarding hypothesis testing, only some of the expected correlations were confirmed (*r* range 0.08-0.96), indicating mixed results.

The *Self-Efficacy in Human-Robot Interaction Scale* [52] was developed to create a German and an English version of a valid and reliable instrument for measuring people’s perceived self-efficacy in dealing with robots. The first version of the Self-Efficacy in Human-Robot Interaction Scale consisted of items that were either adapted from different questionnaires or theoretically generated. An EFA indicated a 2D solution (namely, self-efficacy and loss of control), which showed good internal consistency (Cronbach α=0.945 and 0.864, respectively). A CFA was conducted with the German version of the measure and a different sample; however, it did not reach sufficient structural validity (chi-square–to–df ratio of 5.21 and poor values for the other fit indexes: RMSEA=0.097; CFI=0.84; SRMR=0.055), and a subsequent analysis with reduced items indicated a 1-factor solution and a good model fit (chi-square–to–df ratio=2.98; RMSEA=0.066; CFI=0.95; SRMR=0.029). This result was replicated for the English version. The comprehensibility of the German version was assessed with 6 older adults. Hypothesis testing performed with correlations indicated sufficient values (*r*>0.30); however, we should note that, with only 1 scale, a general self-efficacy measure was close to this value (*r*=0.271 for the German sample and *r*=0.298 for the English sample). The authors also proposed a short version based on results from the EFA. A CFA indicated good fit of the short version for both the German and English samples. In addition, hypothesis testing indicated correlations of >0.30; however, this was true also for 1 scale that the authors used as a discriminant measure.

Nomura et al [53] developed the *Moral Concern for Robots Scale*. A definition or a theoretical background of moral concern was not clearly provided. The Moral Concern for Robots Scale was obtained by adopting items from existing questionnaires. In addition, they created items based on human moral treatment and scenes of possible robot abuse. Through a questionnaire-based survey, the collected data were analyzed using factor analysis, resulting in a 2-factor structure. No fitting statistics were reported. Each dimension indicated good internal consistency (Cronbach α=0.912 and 0.876). Most of the correlations conducted by the authors for construct validity were of <0.30, and 2 dimensions indicated high correlations with the developed measure, namely, “Mental state” and “Social partner”; however, they were not assessed using validated measures, so these results could not properly be considered as evidence of construct validity.

The *HEXACO-60 for Robots* [56] is based on the HEXACO model of personality and proposes that individuals are characterized by 6 domains. The authors adapted the items of the HEXACO-60 original questionnaire [73] addressing “a robot” as the subject of each original item. Even though the construct was clearly described and had a theoretical background, a representative population was not involved in the elicitation of relevant items; thus, relevance was considered indeterminate. The authors performed an EFA that indicated a 4D solution; fitting statistics were not reported.

Akalin et al [57] developed a scale to evaluate the sense of safety and security of robots for older adult care. The authors developed the items after videos of different types of robot interactions were shown to participants; 3 items were based on the Godspeed Questionnaire Series [74]. Definitions of safety and security used to construct items were not clearly reported. The authors calculated the Cronbach α for the 2 dimensions and for each video scenario presented to the participants. All the Cronbach α values reported were of >0.70, indicating good internal consistency of the scales. Factor analysis was performed to identify the most important item associated with the 2 dimensions.

The *Robotic Social Attributes Scale* (RoSAS) developed by Carpinella et al [54] assesses warmth, competence, and discomfort perceived in robots. While the first 2 dimensions were drawn from social psychology, they lacked a clear definition, making it challenging to assess the content of items related to these dimensions. The development of this scale involved 4 studies. In the first study, an EFA was conducted on the Goodspeed Questionnaire Series [74], resulting in 3 factors reflecting anthropomorphism, perceived intelligence, and likeability. In the second study, participants were presented with the Godspeed items, a list of attributes from the stereotype content model, and the Bem Sex-Role Inventory [75,76]. Participants indicated whether each item was associated with robots. EFA reduced the number of items and suggested 3 dimensions: warmth (Cronbach α=0.91), competence (Cronbach α=0.84), and discomfort (Cronbach α=0.82). The third study trialed the developed RoSAS, presenting participants with familiar and unfamiliar animals and human linguistic categories to demonstrate that the dimension of “discomfort” emerges when individuals are evaluating robots. In the fourth study, the questionnaire was validated by comparing different types of robots to assess whether participants’ perceptions varied based on the scale. However, references to support the hypotheses were not provided. In a separate study, Oliveira et al [55] performed a Portuguese translation of the RoSAS and assessed the comprehension of its items. A CFA suggested that the 3 dimensions were a good solution (CFI=0.98; RMSEA=0.05; SRMR=0.06), leading to a reduction in the number of items. Correlations with other measures for construct validity and reliability assessments yielded conflicting results.

The *Rapport-Expectation With a Robot Scale* [63] was designed to measure people’s expectations regarding rapport with robots. To create this scale, students watched science fiction movie clips featuring robots and were asked about their feelings toward interacting with robots, distinguishing between fictional and real robots. Items were developed based on participant responses and from previous research. Subsequently, an EFA was conducted with a small sample, revealing a 2D solution (Cronbach α=0.919 and 0.848). Unfortunately, no fit indexes were reported. To assess construct validity, the same participants were used, with the assumption that there would be variations in their responses based on the different video clips they had viewed. Differences in scores were indeed found, but it is difficult to interpret these results as there was no provided evidence to support the formulated hypothesis. Subsequently, an experimental task was carried out to evaluate predictive validity. However, the results were inconsistent as only 1 of the 2 hypotheses was confirmed.

The *Robot Anxiety Scale* (RAS) [61] was developed to measure anxiety that inhibits people from interacting with robots. The items for this scale were generated through a pilot survey, and content validity was assessed. A subsequent EFA revealed a 3D factor solution. Following this, a CFA indicated a good fit (GFI=0.949; AGFI=0.917; RMSEA=0.066) for each scale (Cronbach α=0.840, 0.844, and 0.796). Construct validity was evaluated by comparing the RAS with 2 other anxiety measures, all showing correlations of <0.30. Cai et al [62] translated the scale from Japanese to Chinese and assessed its comprehensibility and item content validity. Their study included a CFA that confirmed the RAS’s structural validity (chi square–to–df ratio=3.26; SRMR=0.02; CFI=0.99; GFI=0.96; TLI=0.98; RMSEA=0.06), and correlations for construct validity indicated good construct validity (absolute values of *r* ranged from 0.42 to 0.81). Overall, the correlations between the 2 studies yielded mixed results.

The *Frankenstein Syndrome Questionnaire* (FSQ) developed by Nomura et al [67] is a questionnaire to gauge people’s acceptance of humanoid robots. To develop this questionnaire, a survey was conducted to gather opinions, attitudes, and feelings regarding humanoid robots from students in both Japan and the United Kingdom. A group of experts later reviewed the extracted items for content validity. The questionnaire was administered on the web, and a factor analysis revealed a 4D solution (Cronbach α range 0.693-0.909). GFIs were not reported. In a subsequent study, the cross-cultural validity of the FSQ was examined [66], revealing differences in responses between Japanese and UK populations.

Nomura et al [68] developed the NARS to assess the predispositions in behavior or reactions toward robots. They initially gathered opinions through a pilot survey extracting 13 sentences and obtained an additional 20 sentences from 2 other measures. The content validity was confirmed through expert discussions. During the validation, an EFA revealed a 4-factor structure, and a CFA indicated a good fit (GFI=0.900; AGFI=0.856; RMSEA=0.080), with Cronbach α coefficients ranging from 0.648 to 0.782. Construct validity was assessed using Pearson correlation with a measure of anxiety, but all coefficients were of <0.30. Test-retest reliability, assessed using Pearson correlation, showed mixed results: 2 subscales had good reliability (*r*=0.706 and *r*=0.740), but the “Negative attitudes toward emotions in interaction with robots” subscale did not (*r*=0.538). Syrdal et al [71] assessed the NARS in the English population after translating it. They removed 3 items, although the Cronbach α was not reported for each subscale. They conducted a principal component analysis to assess item loadings on each dimension. Construct validity was assessed using 12 personality traits [77], yielding mixed results. A Portuguese validation of the measure was conducted by Piçarra et al [69], resulting in a 2D solution and a good model fit (CFI=0.93; TLI=0.90; RMSEA=0.065). Each subscale showed good internal consistency (Cronbach α=0.73 and 0.75), although construct validity was not evaluated using other standardized measures. The Polish version of the measure, as conducted by Pochwatko et al [70], resulted in a 2D solution with 2 items removed. Both subscales exhibited good internal consistency (Cronbach α=0.84 and 0.79), but the study did not provide sufficient information to assess construct validity. Xia and LeTendre [72] conducted a cross-cultural validation of the questionnaire, recruiting American and international-background students. A CFA confirmed the 3-factor structure (CFI=0.93; TLI=0.91; RMSEA=0.08; SRMR=0.08), and internal consistency was also confirmed (Cronbach α ranged from 0.773 to 0.818). The study revealed differences between the 2 groups of students. It is important to note that the structural validity of the NARS yielded conflicting results, with some studies suggesting a 3D solution whereas others proposed a 2D solution.

The *Perception to Humanoid* scale developed by Kamide et al [58] was designed to assess people’s perspectives when evaluating humanoid robots. University students were required to describe their impressions after viewing a video recording of a humanoid robot. The responses were categorized into groups and adapted into items. An EFA indicated a 5D solution, with each dimension demonstrating good internal reliability (Cronbach α ranging from 0.79 to 0.86). However, no CFA or GFIs were reported.

To assess attitudes toward domestic robots, Ninomiya et al [59] developed the *Multidimensional Robot Attitude Scale*. The authors did not provide a specific definition of “attitude,” and they generated scale items based on descriptions provided by study participants. EFA was conducted, revealing a 12D structure. Subsequently, 2 to 7 items were selected for each factor based on their loadings with the aim of ensuring sufficient differentiation among them. The Cronbach α values for most dimensions exceeded 0.70 except for the value for the “control” dimension, which was 0.643.

The *Social Service Robot Interaction Trust* [48] assesses consumers’ trust in interactions with artificial intelligence social robots. The scale’s items were generated through a literature review process and interviews, subsequently evaluated through a focus group. An EFA revealed an 11-factor solution. The Cronbach α values ranged from 0.82 to 0.94, and a CFA indicated a good model fit (RMSEA=0.03; CFI=0.96; TLI=0.96; SRMR=0.05). Concurrent validity was assessed by comparing the Social Service Robot Interaction Trust with the Interpersonal Trust Scale [78] and the Technology Artifact Scale [79], revealing high correlations (*r*=0.78 and *r*=0.84, respectively).

### Instruments to Assess Older Adults’ Psychological Dimensions Toward Robots

The *Robot-Era Inventory* [42] was designed to measure older adults’ acceptance of social robots across 5 dimensions based on the Robot-Era Model proposed by the authors. The inventory items were derived from existing scales found in the literature. A preliminary validation of the questionnaire was conducted. The internal consistency analysis yielded mixed results, with 2 of the proposed subscales showing insufficient Cronbach α values (0.67 and 0.69). Construct validity was assessed by examining the correlations between the Robot-Era Inventory and the Unified Theory of Acceptance and Use of Technology, although a clear hypothesis was not reported. The associations between the dimensions showed mixed results.

The *Almere model* was developed to test the acceptance of assistive social agents by older adults [21]. The questionnaire items were adapted from the UTAUT to fit the context of assistive robot and screen agent technology, specifically addressing older adult users in a care home environment. Additional constructs were considered, and items were adapted from questionnaires in the literature. A path analysis was used to test hypothesized relationships among the dimensions. In an experiment comparing responses to a robot in more social versus less social conditions, differences were found on 4 subscales. The study showed sufficient internal consistency of the instrument. He et al [65] translated the Almere Technology Acceptance Questionnaire into Mandarin Chinese and evaluated its psychometric properties among older adults in China. They performed a content analysis with 6 experts. EFA followed by CFA revealed a 9D solution (chi square–to–df ratio=2.006; RMSEA=0.069; root mean-square residual=0.059; GFI=0.816; incremental fit index=0.913; TLI=0.896; CFI=0.912). Cronbach α coefficients indicated mixed results, ranging from 0.664 to 0.891, indicating varied internal consistency across dimensions. The test-retest reliability coefficient was satisfactory, with an overall value of 0.980 and domain-specific values ranging from 0.918 to 0.986 [65].

### Instruments to Assess Psychological Dimensions of Health Care Professionals Toward Robots

The *Robot Use Self-Efficacy in Healthcare Work* [60] is a measure developed and validated with health care workers to assess their self-efficacy in using robots in their work. There was no reported information regarding the items’ development. The validation of the measure indicated sufficient internal consistency (Cronbach α=0.90); only factor loadings of the factor analysis performed were reported. Regarding correlations performed for construct validity, results obtained for the 6-item version of the measure were not reported. Instead, the authors performed a correlation analysis for the short version of the measure (3-item version). Most correlations with other measures were insufficient (*r*<0.30) except for one (*r*=0.33), which was measured with only 1 item, and its validation was not reported.

The Chinese version of the *Attitudes Toward the Use of Social Robots* (ATTUSR-C) [49] questionnaire is a modified and translated version of the questionnaire proposed by Costescu and David [80]. Although the original version provides a clear definition of “attitude,” the study was not aimed at validating the questionnaire, and there was no evidence of concept elicitation or literature search in item generation. In this version, a panel of 5 expert academic nursing professors assessed the content validity of the ATTUSR-C questionnaire, rating item clarity and appropriateness. Items with an item content validity index of <70% were eliminated. In addition, 10 clinical instructors assessed the instrument for face validity by evaluating the clarity of each questionnaire item. This process indicated sufficient evidence for relevance and comprehensibility of the items; however, the professionals were not asked about the comprehensiveness of the items, and thus, it was evaluated as insufficient. During validation, the EFA interpretation led to a 1D solution, with no reported fit indexes. The Cronbach α was sufficient (0.84).

Among the instruments designed for health care, we can highlight the *Technology-Specific Expectation Scale–R* [45], which was developed to assess parents’ expectations in health-related robot interactions. The scale consists of items adapted from the work of Alves-Oliveira et al [81], and additional items were created by the authors, organized into 3 dimensions. Principal component analysis was used to determine the item loadings for each dimension; however, fit indexes were not reported. Each subscale demonstrated good internal consistency (Cronbach α=0.869, 0.839, and 0.800). Details regarding the sample used for this analysis were not provided.

## Discussion

### Principal Findings

The use of social robots has generated substantial research interest, and it is unsurprising that numerous studies have explored the variables that influence the human-robot relationship. This exploration is essential for understanding people’s attitudes toward these emerging technological tools. This review aimed to provide both practitioners and researchers with an up-to-date framework of psychometrically validated instruments for assessing the psychological dimensions relevant to the interaction with social and domestic robots. This systematic literature review identified a total of 27 validated measures across 34 articles. These findings suggest a growing interest in psychological constructs related to understanding human-robot relationships, indicating their increasing importance and relevance. Indeed, as detailed in Table 1, the dimensions assessed using the validated scales encompass different constructs.

Although it indicates validated instruments to assess different dimensions, this review also highlights important limitations in terms of psychometric properties. To enhance the quality and accuracy of the available instruments, these limitations should be considered in future development or revisions of instruments for assessing people’s psychological dimensions related to robots. Most of the studies (24/27, 89%) primarily concentrated on assessing the structural validity (12/24, 50% of evidence) and internal consistency (26/27, 96%, of which 18/26, 69% had a high quality of evidence) of the instruments. Construct validity was considered for 63% (17/27) of the instruments (7/17, 41% with a high quality of evidence). Cross-cultural validity was evaluated for only 7% (2/27) of the instruments (both of which exhibited low quality of evidence), and measurement invariance was evaluated for only 4% (1/27) of the instruments (low quality of evidence). Notably, the measurement error and responsiveness aspects were disregarded across all the instruments. Content validity was identified in most of the studies (26/27, 96%); however, none of them exhibited an overall high quality of evidence. Moreover, there was a noticeable scarcity of tools specifically tailored for children, older adults, and health care contexts. This highlights the necessity for the development and validation of instruments encompassing a more comprehensive range of psychometric properties. Such an approach is vital for the advancement of this growing area of research, ensuring that assessments are not only thorough but also tailored to the unique characteristics and needs of diverse populations and contexts.

Regarding content validity, many studies (17/26, 65%) inadequately assessed this property, often demonstrating very low methodological quality and neglecting aspects such as item relevance, comprehensiveness, and comprehensibility. This result aligns with previous findings from other reviews, which have shown that studies often offer unclear definitions of constructs or fail to provide any definition at all [28,29]. Thus, given the interest in validating instruments, the importance of considering this aspect should be further stressed. Content validity refers to how the content of the scale adequately reflects the construct the instrument is intended to measure [82], and it is considered to be the most important measurement property [37]. Relevance and comprehensiveness refer to how well the items are aligned with the construct of interest, ensuring that all key aspects of the construct are thoroughly evaluated [38]. Comprehensibility considers how well items are interpreted, which can have an impact on the quality of responses and measurement accuracy [83]. Therefore, careful consideration of the construct’s definition and its theoretical basis should be taken during the development of the instrument to enhance methodological rigor and improve the quality of the assessment.

Measurement error and responsiveness were not addressed in any of the studies identified. Measurement error indicates the amount of error, systematic and random, that could not be attributed to a true change in the construct measured [82]. It could be assessed through minimally important change, which indicates whether a change in the measurement is considered important [84], or the smallest detectable change, which indicates whether the change in score is of sufficient magnitude to have a low probability of being a random error [85]. Responsiveness indicates how the instrument could detect change over time in the measured construct [86], which is considered to reflect longitudinal validity [87]. However, even if they are important, we should also note that these 2 properties place a strong emphasis in the clinical context [87,88]. Thus, we suggest considering these 2 properties with caution and within the context and aim for which the instrument is used.

Most studies (24/27, 89%) primarily focused on structural validity, typically through EFA or CFA, as well as internal consistency as measured using the Cronbach α. However, it is important to note that several studies (12/24, 50%) had “indeterminate” findings on structural validity and did not report goodness-of-fit statistics for their models or provide sufficient information to assess the appropriateness of their structural models. This result expands upon what Naneva et al [29] previously reported. Thus, the results suggest improvement of the structural assessment of the instruments. It could be suggested to report the goodness of fit in exploratory analysis [89] and further conduct confirmatory analyses to evaluate model fit in relation to this psychometric property. Indeed, while EFA is an exploratory approach to determine the appropriate number of factors, CFA requires a strong empirical foundation and is typically used in later phases on empirical and theoretical grounds [90].

Regarding cross-cultural validity, only 7% (2/27) of the instruments, the NARS [72] and the FSQ [66], assessed this aspect. However, in both cases, there was insufficient evidence to demonstrate cross-cultural validity, and the quality of the methodology was low. Despite the challenges associated with considering this property, cross-cultural validity offers valuable instruments for diverse cultures [91]. Its importance is evident from the multinational studies conducted on the topic of human-robot interactions [92,93] and is also highlighted by the diverse nationalities of the validation samples included in this review. This aspect should be given further consideration in the context of psychological measurements for human-robot relations, with particular attention to the methods used.

Similarly, measurement invariance was examined in only 3% (1/34) of the studies [50]. This is particularly concerning as it would indicate that differences between groups evaluated using most of these instruments could be due to group-specific characteristics rather than to true differences in the dimensions assessed by them [94]. Thus, they should be interpreted with some caution.

Reliability, which refers to the proportion of the overall variance in the measure that can be attributed to true differences between individuals [36], or, in other words, how the variability observed between individuals is not influenced by errors [95], was largely overlooked. Only 15% (4/27) of the instruments provided evidence for the assessment of this property: the Users’ Needs, Requirements, and Abilities Questionnaire; the RoSAS; the Almere model; and the NARS [44,55,65,68]. Consequently, many of the identified instruments did not demonstrate reliability, which represents a significant limitation of most available instruments.

When assessing construct validity, a significant proportion of studies (14/17, 82%) used correlations with other instruments. Nevertheless, these correlations often yielded inconsistent results. A problem faced in this evaluation was that many studies (7/14, 50%) did not establish hypotheses regarding expected correlations beforehand. In the validation of these measures, it is recommended to formulate valuable and clear hypotheses that address the construct under investigation.

Most of the instruments (17/27, 63%) reviewed had a target population of young adults to older adults; however, we should note that they did not consider measurement invariance due to age-related differential item functioning, and thus, it could not be established whether certain items could favor individuals from different age groups with different backgrounds or due to specific response formats [96]. In this regard, this aspect should be considered when developing these instruments. Only 7% (2/27) of the instruments, the Robot-Era Inventory [42] and the Almere model [21], were designed specifically for the older adult population. However, they exhibited limited psychometric properties, indicating the need to develop instruments for this specific demographic group.

Only 11% (3/27) of the instruments considered the clinical context. The Robot Use Self-Efficacy in Healthcare Work [60] and ATTUSR-C [49] were designed for health care professionals, whereas the Technology-Specific Expectation Scale–R focused on parents’ expectations [45]. However, these 3 instruments only demonstrated sufficient internal consistency, indicating that there is still a need to develop psychometrically valid and reliable instruments in the health care context. This is particularly important given the literature’s emphasis on the use of social robots in health care settings [97].

This review indicated that only 19% (5/27) of the instruments in the literature were validated for children. Most of them (3/5, 60%) only demonstrated sufficient structural validity and internal consistency, suggesting that the available measures to assess the psychological dimensions of children toward robots have important limitations. Consequently, there is a need for the development of improved instruments for children.

Finally, it is worth noting that most of the reviewed studies (26/27, 96%) did not effectively use item response theory. While there is some debate regarding the best approach [98], given the conditions of the validation study [99], authors should also take this framework into consideration.

Despite this review indicating a strong interest in developing instruments to assess the psychological facets of the human-robot relationship, it also highlights that only some psychometric properties are systematically considered, whereas other important ones tend to be overlooked. Psychometric properties indicate whether the instrument used is a valid and reliable form to assess the dimension of interest [31]. Poorly or incompletely validated instruments have limited use for specific conditions, populations, and countries [100]. Limitations in these properties may raise concerns regarding the accuracy of reported outcomes in research and in making informed decisions [101].

A significant limitation of the available instruments is the absence of consideration or clear description of context for the robot’s use during development and validation. A precise delineation of the use context is an integral aspect of instrument development and content validity evaluation [38], which serves to indicate the relevance of the developed items composing the instrument. This is important as there is preliminary evidence suggesting that the context in which the robot is presented or used may impact the components of human-robot interaction [29]. This information is critical for practitioners and clinicians as it indicates the appropriate use of these instruments for specific purposes and clinical populations.

This suggests a need to develop instruments with a broader range of psychometric properties through studies with higher methodological quality of evidence. The analysis suggests that, in addition to the commonly assessed psychometric properties, particular attention should be paid to content validity, cross-cultural validity, reliability, measurement invariance, and construct validity through rigorous methodologies. Particular attention should be paid to targeted groups and the potential application of the instruments in different contexts. Measurement error and responsiveness remain important properties, and their assessment should be guided by the rationale of the developed instrument. Researchers should consider that these properties have significant weight in a clinical context.

While this study yielded intriguing results, it is important to acknowledge its limitations. The review and analysis in this study primarily focused on questionnaires suitable for assessing social and domestic robots. However, it is crucial to note that questionnaires tailored for other specific types of robots, such as industrial robots, exist and warrant evaluation to offer valuable insights into those domains as well. In addition, there are alternative measures in the existing literature for assessing psychological constructs in the context of human-robot interaction. In this regard, it is worth mentioning the Godspeed Questionnaire Series [74]. The search strategy in this review focused on studies dedicated to the validation of instruments, considering eligible those that addressed this aspect as one of their primary objectives. In the context of the relatively novel field of instrument development for human-robot interactions, it was not feasible to identify gold standards for assessing criterion validity in this review. Nevertheless, it is worth emphasizing that the findings of this study may contribute to the identification of gold standards for other instruments in the future. Indeed, there is significant variability in their use, with some scales being rarely used whereas others are more commonly used (eg, the Almere model) in the literature. The cause of this variability cannot be definitively determined. Notably, the NARS, the Almere model, and the RAS are the oldest scales identified in the systematic literature, potentially contributing to their continued use. This raises the possibility that certain important constructs may be systematically overlooked or the psychometric properties of the instruments might be disregarded. The primary objective of this review was to offer a comprehensive overview of the instruments available to measure various dimensions and conduct a critical selection based on the currently available psychometric properties of these instruments. As another aspect to consider, individuals may have distinct preferences regarding various physical characteristics of robots, and these preferences are likely influenced by personal factors. However, the extent to which appearance can impact or enhance the human-robot relationship remains a topic that requires more comprehensive exploration. Indeed, determining the ideal form that an agent, such as a robot, should take is particularly challenging [102].

### Conclusions

Numerous psychometrically validated instruments exist for assessing various psychological constructs within the realm of human-robot relationships applicable to both social and domestic robots. This review aimed to provide a comprehensive overview of these instruments, offering insights into their psychometric properties. While there is a notable interest in developing and validating such instruments, this review also puts forth guidelines and considerations for both the creation of new instruments and the review of existing ones. This review indicates the necessity to develop and validate new instruments for human-robot interactions encompassing more methodologically rigorous approaches and a broader spectrum of psychometric properties. Researchers should carefully consider the targeted populations and the context of use during development.

## Appendix

Multimedia Appendix 1 Search strategy for each database.

Multimedia Appendix 2 PRISMA checklist.

## Abbreviations

AGFI: Adjusted Goodness of Fit Index
ATTUSR-C: Chinese version of the Attitudes Toward the Use of Social Robots
CFA: confirmatory factor analysis
CFI: comparative fit index
COSMIN: Consensus-Based Standards for the Selection of Health Measurement Instruments
EFA: exploratory factor analysis
FSQ: Frankenstein Syndrome Questionnaire
GFI: goodness-of-fit index
NARS: Negative Attitudes Toward Robots Scale
PRISMA: Preferred Reporting Items for Systematic Reviews and Meta-Analyses
RAS: Robot Anxiety Scale
RMSEA: root mean square error of approximation
RoSAS: Robotic Social Attributes Scale
SRMR: standardized root mean square error
TLI: Tucker-Lewis index

## Data Availability

Data sharing is not applicable to this paper as no data sets were generated or analyzed during this study.
