# Supplementary material for: Instruments for Measuring Psychological Dimensions in Human-Robot Interaction: Systematic Review of Psychometric Properties
Source: J Med Internet Res. 2024 Jun 5;26:e55597. doi: 10.2196/55597 (PMC11187516; doi:10.2196/55597)
Supplement: Multimedia Appendix 1 [file jmir_v26i1e55597_app1.docx]

*Supplementary Material*

Appendix A. Research strategy for each database.

| Scopus (N = 1786) | TITLE-ABS-KEY ( ( robot AND ( social OR home OR domestic ) OR human-robot ) AND ( scale OR survey OR questionnaire OR measur* OR assessment ) AND ( psychom* OR valid* OR reliab* ) ) |
| --- | --- |
| PubMed (N = 147) | (robot[tw] AND (social[tw] OR domestic[tw] OR home[tw]) OR human-robot[tw]) AND (scale[tw] OR survey[tw] OR questionnaire[tw] OR measur*[tw] OR assessment[tw]) AND (psychom*[tw] OR valid*[tw] OR reliab*[tw]) |
| IEEEXplore (N = 1895) | (("Abstract":"robot" AND ("Abstract":"social" OR "Abstract":"home" OR "Abstract":"domestic")) OR "Document Title":"Robot" OR "Abstract": "human-robot") AND ("All Metadata":"scale" OR "All Metadata":"questionnaire" OR "All Metadata":"survey" OR "All Metadata":"measur*" OR "All Metadata":"Assessment") AND ("All Metadata":"psychom*" OR "All Metadata":"valid*" OR "All Metadata":"reliab*") |
